# Genotyping of Commercial European *Cannabis* Seeds Based on Multiple Mapped Marker Loci: A Comparative Study of Drug and Hemp Varieties

**DOI:** 10.3390/plants14193050

**Published:** 2025-10-02

**Authors:** Marcello Borin, Francesco Scariolo, Maddalena Cappello Fusaro, Irene Lucchetta, Gio Batta Sacilotto, Marco Gazzola, Stefano Bona, Gianni Barcaccia

**Affiliations:** 1 Gruppo Padana Ortofloricoltura S.S., Via Olimpia 41, 31038 Treviso, Italy; marcello.borin@gruppopadana.com (M.B.); giobatta@gruppopadana.com (G.B.S.); marco.gazzola@gruppopadana.com (M.G.); 2 Department of Agronomy, Food, Natural Resources, Animals and Environment (DAFNAE), University of Padova, Campus of Agripolis, Viale dell’Università 16, 35020 Padova, Italy; francesco.scariolo@unipd.it (F.S.); maddalena.cappellofusaro@unipd.it (M.C.F.); stefano.bona@unipd.it (S.B.)

**Keywords:** SSR genotyping, *Cannabis sativa*, drug-type *Cannabis*, cannabinoids

## Abstract

*Cannabis sativa* L. (2n = 2x = 20) is a widely recognized species within the Cannabaceae family. Despite its utilization for medicinal, recreational, and industrial purposes, alongside its extensive historical background, the number of genetic and biotechnological studies of this plant species has decreased due to legal ramifications and prohibition campaigns associated with its use and cultivation. For many years, the development of novel varieties has been pursued solely by cultivators, as domestic growers have transitioned their work from cultivation to breeding *Cannabis* lineages. Recently, the application of genomics has facilitated a surge in methodologies aimed at marker-assisted selection, germplasm management, genetic differentiation, authentication of cultivated varieties or cultivars, and forensic applications such as safeguarding intellectual property rights. Nevertheless, the utilization of molecular markers for the advancement of commercial varieties through marker-assisted breeding (MAB) frameworks remains rare. This investigation was designed to evaluate a previously established informative microsatellite (SSR) array for the genotyping of drug-type *Cannabis sativa* cultivars derived from seeds of European origin. A total of 171 samples from 20 varieties were collected from European distributors and analyzed for genetic uniformity and population structure. The results were then compared with previously analyzed hemp samples and drug-type samples of Canadian origin, revealing the identification capabilities of our SSR genotyping method.

## 1. Introduction

*Cannabis sativa* L. is an agronomic plant species of substantial interest because of its numerous applications in the recreational, therapeutic, and industrial domains [1]. This plant can be cultivated to yield fibers [2] (utilized to fabricate various textiles), seeds (abundant in unsaturated fatty acids for consumable oils), and pharmaceutical metabolites derived from its female inflorescences, which are used to synthesize cannabinoids [3] (substances exhibiting psychotropic and psychopharmaceutical properties). *Cannabis* plants can also be used for phytoremediation of contaminated soils thanks to their high biomass yield and ability to retrieve heavy metals from polluted grounds [4]. Among the metabolites synthesized by *Cannabis* plants, delta-9-tetrahydrocannabinol (Δ9-THC, or simply THC) is the predominant psychoactive species, the concentration of which underpins the differentiation between hemp and drug (marijuana) varieties, with the former and latter containing a low concentration of THC (<0.3% by dry weight and therefore nonpsychoactive) and up to 30% THC by dry weight, respectively [5,6].

The genus *Cannabis* belongs to the family Cannabaceae (order Rosales). Its botanical classification has experienced a notably tumultuous path regarding whether the genus was mono- or polytypic since the Linnaean system was introduced [7,8,9]. In 1597, John Gerarde [10] initially delineated the plant species as dioecious, monoecious, and hermaphroditic varieties had yet to be identified [8,11,12]. These different biological variants are commonly observable and prevalent in fiber varieties [8]. Moreover, *Cannabis* also displays sexual dimorphism, with male plants frequently characterized by a reduced crop cycle and increased stature, suitable for fiber production. Furthermore, Small and Cronquist [8] proposed a distinctive taxonomic system that remains widely acknowledged on the basis of two subspecies of *C. sativa*—*C. sativa* subsp. *sativa* and *C. sativa* subsp. *Indica*—with the former being generally taller with narrow leaves and the latter being shorter with broader leaves. Notwithstanding the many and controversial taxonomic classification hypotheses, the *Cannabis* genus is most likely indigenous to western or central Asia, despite being widely distributed and having adapted to different environments globally [13,14]. Hemp typology, initially grown for fiber, was first introduced to western Asia and Egypt and subsequently to Europe between 1000 and 2000 BC, with its cultivation in Europe becoming widespread after 500 AD [14].

Recently, the application of genomics has facilitated the development of methodologies directed toward breeding, germplasm management, authentication of cultivated varieties, and forensic applications, including the safeguarding of intellectual property rights. For *Cannabis*, beyond traditional phenotypic characterization [15], genomic tools such as molecular markers have been extensively employed to investigate the genetic diversity, geographical provenance, and genetic relationships among cultivated germplasms; to characterize varieties; to determine sex; and to construct genetic linkage maps and genomic assemblies [16,17,18,19,20,21,22]. Among these tools, Simple Sequence Repeats (SSRs) stand out for their advantageous features, including their high reproducibility and codominant inheritance. Furthermore, unlike alternative markers such as Single Nucleotide Polymorphisms (SNPs), microsatellites do not require high-throughput technologies or extensive computational resources for their development and evaluation [23,24,25,26,27].

Microsatellites have been extensively employed in *Cannabis* cultivars and genotypes to assess genetic diversity [28,29,30]. Although preliminary investigations have identified and characterized a limited number of polymorphic SSR loci specific to *Cannabis sativa* [31,32,33], these markers have recently been utilized to distinguish between drug- and fiber-type (hemp) varieties [28,32,34,35,36,37,38,39,40,41,42], demonstrating their efficacy as a viable alternative to biochemical analyses [1,43]. Adopting this kind of molecular markers, with respect to geographical provenance, several studies have indicated that the highest genetic diversity is observed within hemp varieties compared to drug varieties, highlighting the possible differentiation of distinct gene pools in Europe and Central Asia [35,44,45,46].

In recent decades, several scientific studies have focused on determining the genetic population structure of the *Cannabis sativa* varieties available on the market, even if their precise geographic origin is essentially unattainable considering their cultivation history. Indeed, following World War II, *Cannabis* cultivation and use were prohibited in many countries and continents, initiating a phase of illegal spread, with amatorial breeders developing new illegal and noncertified varieties [9,14,47,48,49]. The illegal trafficking of drug-type *Cannabis sativa* (marijuana) for recreational use has significantly increased since the 1960s. Most clandestine producers from Africa, Asia, and Latin America seeded the main consumer centers in Europe and North America, where local cultivation and breeding strategies feature new agricultural and distribution methodologies to avoid legal detection.

The production of hybrids between narrow-leaf drug-type *Cannabis* (with a high THC–CBD ratio) and broad-leaf drug-type *Cannabis* plants (with a moderate to high THC–CBD ratio) has also increased worldwide, and vegetative indoor cultivation of these has proliferated. Consequently, the global genetic distinctiveness of drug forms has undergone a significant reduction compared to that in the 1970s and 1980s, when different biotypes were cultivated by traditional farmers in isolated geographic environments [47].

This study aims to analyze the genetic structure of a European drug-type *Cannabis* germplasm collection (171 samples), mainly composed of commercialized varieties available in the market. Furthermore, the present study aims to assess the population structure and individual relationships among the considered accessions, evaluate the effectiveness of the adopted SSR markers in distinguishing drug-type from hemp-type samples, and compare the obtained results with previous datasets developed in early studies on hemp-type populations [30,50].

## 2. Materials and Methods

### 2.1. Cannabis Plant Cultivation

*Cannabis sativa* seeds, including 20 distinct drug-type varieties of significant breeding value, were acquired from eight European distributors and derived from multiple seed companies. Seeds from each variety were grown at the University of Padua (DAFNAE—Department of Agronomy, Food, Natural Resources, Animals and Environment, Campus of Agripolis, Legnaro, Italy) and adopted to sample the leaf material for use in the genomic analyses. From the 20 purchased varieties, 9 to 15 seeds per variety were planted, totaling 244 seeds.

Seeding was initiated on 2 November 2023. Rockwool plugs were used as the germination substrate after being presoaked for 24 h in an acid solution (pH of 5.5) for pH adjustment. The trays were placed inside 120 × 120 cm growth boxes and maintained at approximately 24 °C with a humidity level of 75%. For the first four days, the trays were kept in the dark to promote germination, after which the lights were turned on for periods of 18 h light/6 h dark. Lighting was provided by two Cosmorrow LEDs (Secret Jardin–Agomoon, Manage, BE) (20 W, L50 cm), which delivered a PPF of 54 µmol/s. The trays were manually irrigated with deionized water for approximately four weeks, resulting in the germination of 171 seeds.

After this period, each plant was transplanted into a 3 L pot filled with coconut fiber and transferred to a larger grow-box equipped with a hydroponic irrigation system. In this environment, the humidity was gradually reduced to 55%. Lighting was provided by Valoya RX325 Solray385 lamps (Valoya Inc, Buffalo, NY, USA), which delivered a PPF of 870 µmol/s with an 18/6 h photoperiod, and the plants were automatically watered twice daily for 2 min per session. The nutrient solution, which was prepared by Canna Vega A + B, had a pH of 5.8 and an electrical conductivity (EC) of 0.8 S/cm for the first 10 days, then increasing to 1.2 S/cm. The nutrient solution parameters and the irrigation regime were controlled by NIDO, an automated system for managing hydroponic cultivation settings.

### 2.2. Cannabis Sampling and DNA Extraction

During cultivation, a total of 171 plants were germinated and then sampled and analyzed. Fresh leaf samples from these 171 individuals were collected in January 2024 and preserved at −20 °C. Further information regarding the origin, characteristics, and typology of the analyzed samples is reported in Supplementary Table S1.

Genomic DNA was extracted from 70 to 100 mg of each sample using a DNeasy 96 Plant Kit (Qiagen GmbH, Hilden, Germany) following the manufacturer’s instructions. The quality of the genomic DNA samples was assessed by electrophoresis on a 1% (*w*/*v*) agarose gel stained with 1X SYBR Safe™ DNA Gel Stain (Life Technologies, Carlsbad, CA, USA) in a Tris–acetate-EDTA (TAE) running buffer. The yield and purity were evaluated using a NanoDrop 2000c UV–vis spectrophotometer (Thermo Scientific, Pittsburgh, PA, USA). Following DNA quantification, all the DNA samples were diluted to a final concentration of 20 ng/μL for use as templates for PCR amplification.

### 2.3. Analysis of SSR Marker Loci

Amplification reactions were performed using the three-primer strategy reported by Schuelke [51] with some modifications. Additionally, 20 primer pairs were organized into 4 multiplexes and used to amplify all 171 genomic DNA samples using the method and protocol described in Borin et al. [30] with no modification. Consistent with the multiplex PCR method, the fragment length estimation and polymorphism identification were also conducted according to the previous study. Organization of primers into the four adopted multiplexes is reported in Supplementary Table S2.

Moreover, genotyping data derived from 104 individual plants belonging to 11 distinct hemp cultivars presented in our preceding publication [30] were compared with these new findings, alongside 10 samples from drug-type Canadian varieties previously assessed with identical SSR marker analysis [50]. Further information regarding the origin, characteristics, and typology of the analyzed samples are reported in Supplementary Table S1.

### 2.4. Molecular Data Analysis

The population structure of the *C. sativa* collection was investigated using the model-based (Bayesian) clustering algorithm implemented in STRUCTURE software v.2.2 [52], which groups individuals according to marker allele combination and distribution during multiple runs. Each population was characterized by the frequency of alleles at each locus, and the individuals were assigned to one or more populations.

The number of founding groups depended on the two analyses conducted: the range was set from 1 to 25 for the 171 samples from 20 drug-type varieties of European origin and from 1 to 40 for the 285-sample dataset, including 171, 10, and 104 drug-type varieties of European origin, drug-type samples from varieties of Canadian origin, and hemp-type cultivars of European origin, respectively.

The method described by Evanno et al. (2005) [53] was used to evaluate the most likely estimation of K. A burn-in of 2·10^5^ and a final run of 10^6^ Markov chain Monte Carlo (MCMC) steps were set for both analyses.

Statistical analyses for all SSR marker loci were performed using the PopGene software package v. 1.32 [54]. The observed number of alleles per locus (na), Levene’s observed heterozygosity (Ho; [55]), Nei’s expected heterozygosity (He; [56]) and average heterozygosity (Ha; [56]) were computed for each SSR locus and overall SSR markers.

The genetic similarity (GS) in all the pairwise comparisons was computed using Rohlf’s coefficient of simple matching and conducted under default settings by the NTSYS software package v. 2.21c [57].

Moreover, the obtained dataset was adopted and imported into the R environment for further analyses. A *genind* object was created using the *adegenet* package [58,59] in RStudio software version 2025.05.0+496 (https://posit.co/products/open-source/rstudio/, accessed on 1 October 2025), and a discriminant analysis of principal components (DAPC) graphical representation was plotted using the ggplot2 package [60]. The *aboot* command from the *poppr* package [61] was used to create a UPGMA dendrogram on the basis of Nei’s genetic distance with [56] 1000 bootstrap replicates. Finally, the *pheatmap* function was used to create a genetic similarity-based heatmap using the previous results from NTSys software v.2.10e.

Private allele frequency in all SSR loci between the drug- and hemp-type populations was also calculated via GenAlEx 6.5, a genetic analysis add-on for Microsoft Excel [62].

## 3. Results and Discussion

After the successful cultivation, collection, and data analysis of 171 *Cannabis sativa* samples from plants derived from European distributors, all samples were investigated using our previously developed SSR marker-based genotyping panel. After earlier testing was conducted on 11 hemp cultivars, the markers had demonstrated success in assessing heterozygosity/homozygosity, genetic uniformity, stability, and genetic variation within and among cultivars [30].

In our previous studies, 301 alleles were detected among 11 cultivars, with several observed alleles per locus, ranging between 3 (SSR_6-4) and 28 (SSR_X-1). According to Botstein et al. (1980) [63], 16 of 20 SSR loci were highly informative in terms of their polymorphism information content, which was above 0.5 (PIC > 0.5), and 4 SSR loci were reasonably informative (0.5 > PIC > 0.25). The number of polymorphic loci was high among all analyzed cultivars [30].

Using the results gathered from our genotyping method on 11 hemp cultivars and 10 drug-type samples of European [30] and Canadian origin [50], respectively, we proceeded with the present study of 171 individuals representing 20 different drug-type varieties from European seeds. All 285 samples from the described different sources were combined, and a grand total of 403 alleles were detected from the 20 loci investigated. The number of alleles per locus fell between 7 (SSR_6-4) and 34 (SSR_X-1), averaging at 20.15 ± 7.77. The results revealed that the marker set adopted was not only functional but also informative for the drug-type varieties analyzed.

### 3.1. Genetic Analysis of 171 Drug-Type Cannabis Samples from European Seeds

Adopting the selected SSR markers, the first objective was to study the genetic structure of the germplasm collection, consisting of 171 drug-type samples of European origin, and the obtained dataset was investigated to infer the population structure and relationships among individuals. This analysis was conducted to confirm the claimed variety labeling of the 20 varieties acquired from European distributors from 7 distinct vendors, and to do so, the population structure of the *C. sativa* collection was investigated with STRUCTURE software v.2.2 [52].

**Figure 1:**
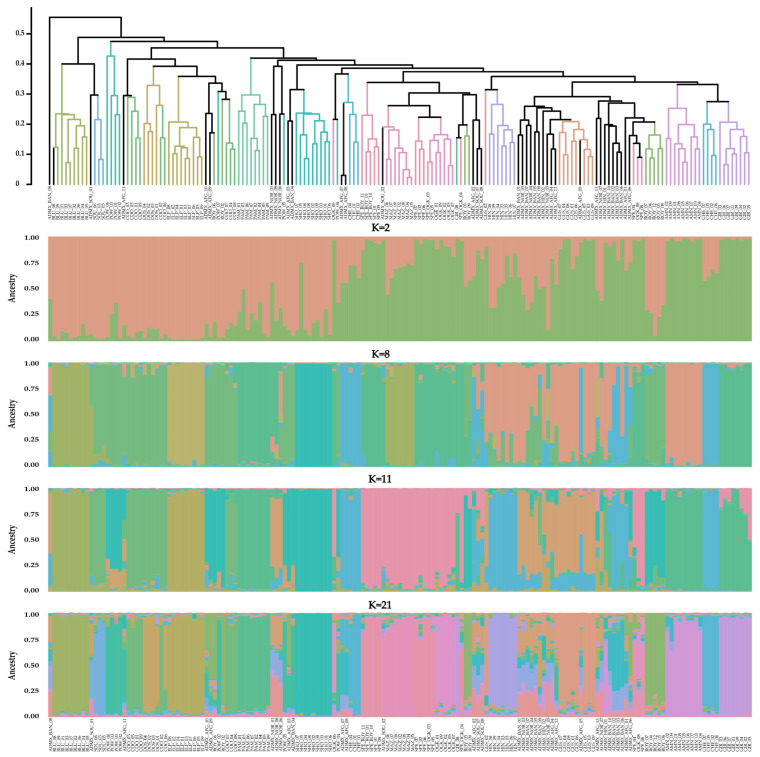
Combined UPGMA–STRUCTURE with K = 2, K = 8, K = 11, and K = 21 values of the 171 *Cannabis* accessions from 20 varieties acquired from European distributors based on the developed SSR marker loci genotyping system. Variety IDs can be found in Supplementary Table S1.

Following the procedure of Evanno et al. (2005) [53], the most informative ΔK values were found to be at K = 2, K = 8, K = 11, and K = 21, the results of which are shown in Figure 1, ordered by a genetic distance-based UPGMA dendrogram.

This analysis shows that the majority of samples were clearly divided on the basis of variety. At K = 21, 17 varieties were clearly represented by 17 distinct and stable genetic populations.

For K = 21, all 135 samples assigned to a unique variety shared, on average, 86.89 ± 10.90% identity with their respective population cluster, with the highest sample identities being found for GIR (94.12 ± 1.92%) and BLU (93.41 ± 2.80%); three other varieties (ELP, MAZ, and SHO) were also highly homogeneous, with memberships above 90%. Notably, samples from these highly homogeneous varieties were consistently placed in the same population cluster at all the tested K values (2, 8, 11, and 21) and always had high ancestral membership.

Moreover, five samples, namely three ROY and two OGK samples, shared a much greater identity with the SPE population than with their corresponding varieties, as also observed for all K values. These plants were therefore considered part of the SPE variety, as further discussed in Section 3.2.

The samples from the remaining three varieties, namely AFG, BAN, and NOR, instead presented mixed population identities. Additionally, two and four samples from the HIN and SOU varieties, respectively, had similar unclear classifications. Further insights and considerations into these samples are given in Section 3.2.

**Figure 2:**
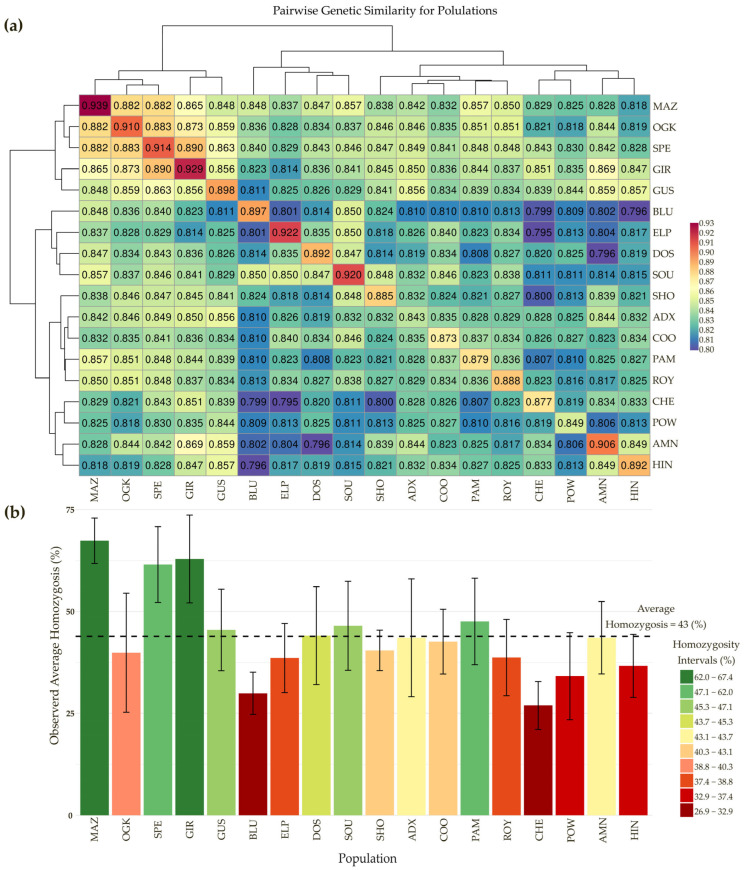
(**a**): Genetic similarity (GS) matrix for the 171 purchased samples from 20 varieties clustered into 17 populations and 1 admixed group (ADX) representing all the non-assigned samples; the internal GS is shown in the diagonal cells, and the GS between populations in the intersections. (**b**): For each genetically distinct population and the admixed group (ADX), the average observed homozygosity (Hom) is also reported as an average with the respective standard deviation. Variety IDs can be found in Supplementary Table S1.

The UPGMA dendrogram results for these samples are similar and, in most cases, identical to those from the K = 21 STRUCTURE analysis. All unclassified samples with no clear identification into a single population associated with the acquired varieties were therefore labeled “admixed” in the subsequent analyses. These samples shared an average identity of only 37.15 ± 17.38% with their highest related population, and identity assessment was not possible. After classifying all the samples into 17 distinct populations and 1 admixed group, all the clusters were investigated for their internal and external genetic similarities. These results are illustrated in Figure 2, where the genetic similarity (a) and observed homozygosity (b) are described for all populations.

The genetic similarity calculated within varieties (shown in the diagonal cells in Figure 2a) was found to be the highest in the GIR, MAZ, and ELP varieties, where it was on average higher than 92%, while, as expected, the group with the lowest internal similarity was the admixed group (ADX), with an average GS value of 84.26 ± 3.23%.

The average observed homozygosity was calculated for each genetically distinct population, and the admixed group was highly variable, with highly homozygous samples and very heterozygous varieties, such as MAZ with 67.38% ± 5.58 homozygosity and CHE with 26.93% ± 5.87 homozygosity, respectively. On average, homozygosity was 43.90% across all the samples.

**Figure 3:**
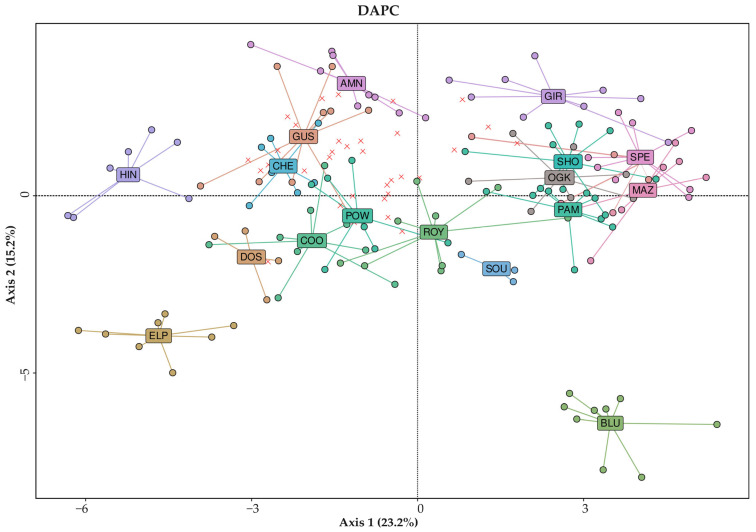
DAPC displaying the centroids of individual plants across all European-origin drug-type *Cannabis* varieties plotted according to the first two dimensions on the basis of the 20 SSR marker loci genotyping method. Labeling: X = admixed samples, ○ = samples assigned to a stable genetic population. Variety IDs can be found in Supplementary Table S1.

In Figure 2, information regarding the close relationships between different varieties is also reported. For example, genetic similarity between populations was at its highest between the MAZ, OGK, SPE, and GIR samples, as also clearly highlighted in Figure 3, where these samples clustered together in the top-right area of the DAPC graph.

From the DAPC graph, clear differences in the genetic structure of the analyzed populations can be observed, with few varieties (BLU, HIN, GIR, AMN, and ELP) having most of their samples grouped together and distinguishable from all others. The remaining ones were mostly mixed and overlapped with accessions from other varieties even when grouped together, likely due to the use of only two plot coordinates.

As previously mentioned, genetic variability within European varieties was observed to be low, owing, as expected, to the fact that drug-type plants underwent high levels of crossbreeding and recombination-related events for commercial purposes [47]. Although this is a mere hypothesis, further comparative analyses, including ancestral germplasm (e.g., landraces), could more directly demonstrate the extent of genetic diversity loss among the European varieties of *Cannabis*.

### 3.2. Labeling Concerns with the Acquired European Seeds

As previously stated, various concerns have been raised regarding the quality and genetic identity of the acquired varieties due to the obtained genotyping results. The main critical outcomes were as follows:

(1) Numerous samples, especially those from the AFG, BAN, and NOR varieties, had no clear population identity;
(2) Seeds labeled as ROY and OGK were found to be part of the SPE population rather than their respective populations (like the majority of their respective samples);
(3) Samples from the HIN varieties were found to be not only genetically but also phenotypically distinct, despite belonging to the same batch of acquired seeds.

Regarding the first outcome, our genotyping analysis clearly indicated that some varieties could not be defined, thus respecting the DUS parameters [64,65], as some samples had mixed and variable population ancestry with very low genetic similarities among them. We hypothesized that the seeds in question were derived from uncontrolled pollination events between various parental plants and were then labeled as varieties and subsequently sold by vendors.

Concerning the second point, three and two seeds from the ROY and OGK varieties, respectively, were found to be associated with the SPE population cluster. On average, these samples shared 84.36 ± 14.85% identity with the SPE population, with their identity to the supposed original variety being negligible, at only 0.67 ± 0.81% and 1.90 ± 1.70% for the ROY and OGK samples, respectively. These findings suggest that errors were made during the seed collection or packaging process, as all these seeds were produced and sold by the same vendor, thus likely resulting from seeds from a variety (probably SPE) being incorrectly incorporated into the ROY or OGK packages.

Finally, regarding the third issue, two of the nine samples from the HIN variety were completely genetically distinct from the others, and their distinctiveness was also phenotypically detected in the later stages of vegetative and flowering development. Indeed, for this variety, the seven genetically related samples (which shared 88.00 ± 3.87% identity with the population and exhibited 89.19 ± 2.46% internal genetic similarity) were the only ones observed to display autoflowering behavior during their growth at 18 h of light per day. *Cannabis* autoflowering varieties are insensitive to the photoperiod for flowering initiation, which is commonly induced at 12 h of light per day in all photosensitive varieties [66]. Interestingly, the two genetically distinct samples from HIN only shared 22.90% and 10.60% identity, respectively, with their supposed variety and, furthermore, displayed no autoflowering behavior during their cultivation, even if subjected to the same agronomical conditions as the others. Owing to these findings, the two outliers were assumed to be derived from different varieties, which were probably not included in the present study, and to be incorrectly labeled and/or packaged in the HIN variety.

### 3.3. Discriminating Between Drug- and Hemp-Type Samples

After genotyping and analyzing the population structure of the studied drug-type samples, this study aimed to verify the ability of the adopted SSR method to discriminate between drug- and hemp-type *Cannabis* samples and to investigate the genetic relationships between these samples and other samples from two past studies conducted by Borin et al. (2021) [30] and Borin (2024) [50]. These two studies considered 11 hemp-type *Cannabis* varieties (104 samples) and 10 samples from drug-type varieties of Canadian origin, respectively.

First, as previously conducted with the European drug-type dataset, the population structure of the complete *C. sativa* collection (285 samples) was investigated using STRUCTURE software v.2.2 [52].

**Figure 4:**
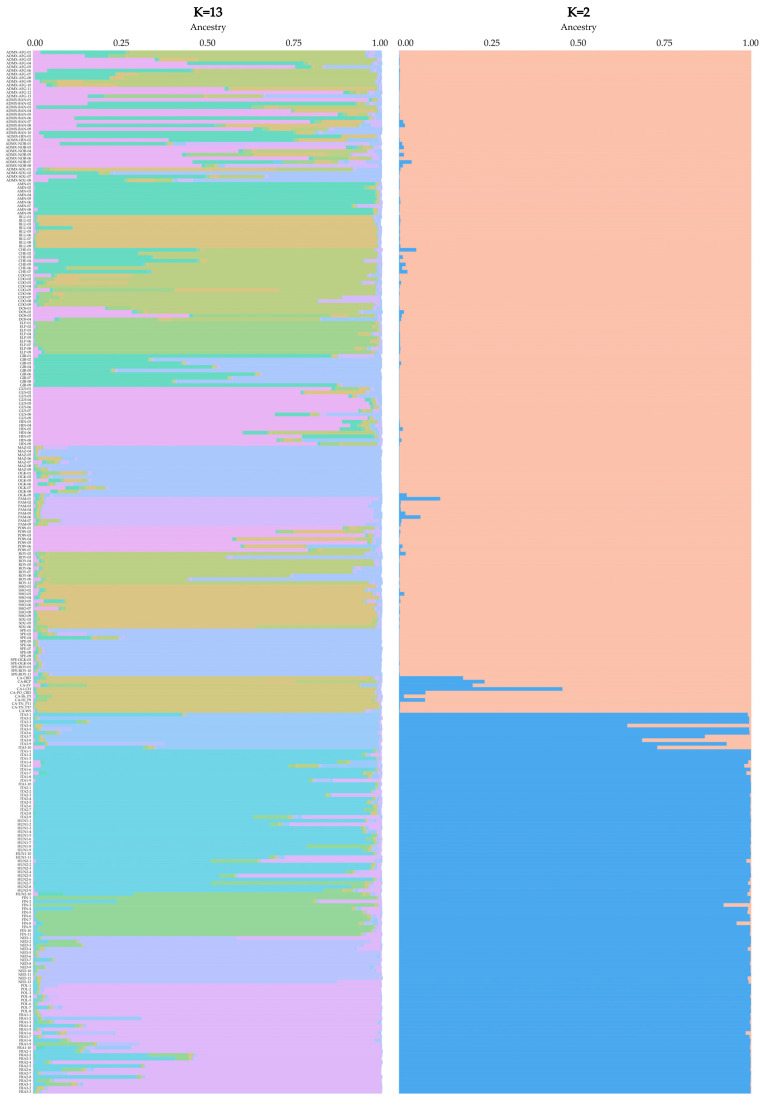
Population structure with K = 2 (**right**) and K = 13 (**left**) of all (285) *Cannabis* accessions, including 171 European-origin and 10 Canadian-origin (CA) drug-type samples and 104 European-origin hemp-type samples. Variety IDs can be found in Supplementary Table S1.

This analysis provided the most informative ΔK values for K = 2 and K = 13, with the specific results reported in Figure 4.

The analysis of the complete dataset clearly differentiated *Cannabis* accessions into THC-drug- and hemp-type accessions, as shown in Figure 3 for the K = 2 clustering. All the drug- and hemp-type samples (labeled in blue) exhibited, on average, 98.85 ± 4.50% and 98.46 ± 5.39% membership to different clusters, respectively, clearly indicating the capacity of the adopted SSR marker set to assign unknown *Cannabis* samples to the drug or hemp typologies.

Afterward, the K = 13 division exhibited that it was able to classify samples into their respective, or related, variety or cultivar groups, as shown in Figure 4 (left).

By dividing the 285 *Cannabis* samples into 13 ancestral populations, with 8 and 5 entirely comprising drug- and hemp-type samples, respectively, no significant membership of hemp-type ancestral identity to the drug-type clusters or vice versa was observed.

On average, drug-type samples exhibited lower identities with their respective populations, with an average membership of 79.66 ± 19.79%, which was lower than that of the hemp-type genotypes (86.38 ± 14.72%), even when not considering those labeled as admixed for the drug-type group (83.62 ± 18.55% identity in this case).

**Figure 5:**
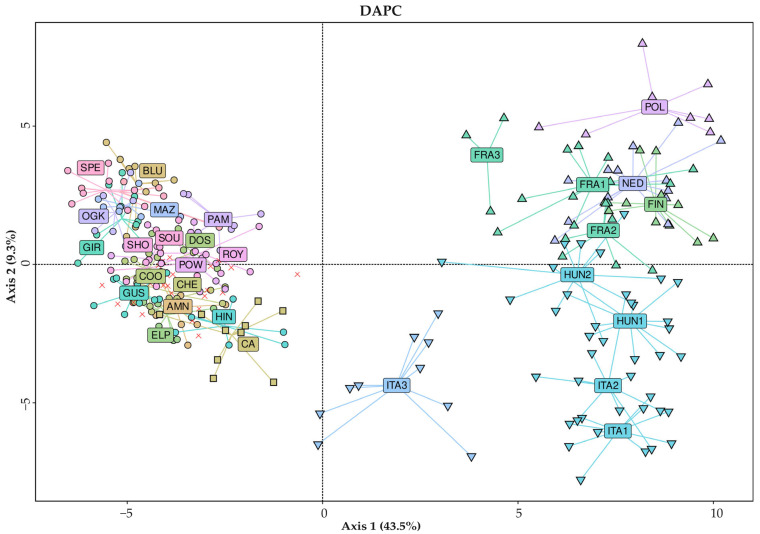
DAPC displaying centroids of individuals for all 285 analyzed samples. Labeling: X = admixed samples, ○ = THC drug-type variety of European origin, □ = THC/CBD drug-type variety of Canadian origin, ▽ = hemp-type cultivars of Italian/Hungarian origin (southern European), △ = hemp-type cultivars of Polish, French, Finnish or Dutch origin (northern European). Variety IDs can be found in Supplementary Table S1.

With the same dataset and while keeping the assignment of samples to populations as shown in the STRUCTURE analysis, a DAPC was conducted, with a graph being produced to visualize the relationships between the identified populations, as described in Figure 5.

All the samples analyzed (hemp and drug types) are included in the DAPC plot in Figure 5. Drug-type varieties (squares and circles in the graph) were genetically distant and distinguishable from all hemp cultivars (up and down triangles), confirming the expected clustering and previous findings [34,67,68].

All drug-type samples were clearly placed in the left portion of the plot, where none of the hemp-type samples were present.

Most identified populations also had a clear position within the graph, with the population composed of Canadian samples (light brown squares), for example, being in the middle cluster and being distinguishable from the rest of the samples.

With respect to the hemp-type samples, an evident distinction was observed between accessions from southern European cultivars (Italy and Hungary) and those from northern European cultivars (Poland, France, Finland and The Netherlands), represented by downward triangles in the bottom right quadrant and upward triangles located in the top-right quadrant, respectively.

Unfortunately, owing to the complicated history of drug-type varieties, as addressed in the Introduction [47,48], besides clearly distinguishing between drug-type samples of Canadian or European origin, the present study could not provide reliable insights into the precise geographical origin of the European samples considered, with no certified information provided for any of them.

Finally, the presence of private alleles among the SSR loci adopted was verified to investigate their potential ability to discriminate between drug- and hemp-type populations.

Overall, of the 403 polymorphisms identified from the 20 selected SSR loci, only 322 had at least 10% frequency in the two macro-groups studied. Among these, 49 polymorphisms were found to be at least five-fold more common in one population than in the other (Supplementary Figure S3) and could therefore be considered suitable candidates for future classification of unknown samples into drug- or hemp-type groups.

Further studies on the frequency and distribution of private alleles could provide valuable insights into the most effective loci for various applications [69]. In the present case, private alleles aid in distinguishing drug- from hemp-type populations. More broadly, these alleles could support variety authentication, genetic traceability, and the optimization of breeding programs in this species and typology.

## 4. Conclusions

Owing to these findings, the present study demonstrated the effectiveness of the SSR marker set adopted for assessing the genetic distinctiveness, uniformity, and genetic variability of individual varieties and for estimating heterozygosity/homozygosity statistics of single plants or populations. The extent of genetic variation within different *C. sativa* varieties and their genetic relationships were also revealed. The resulting information may serve as a useful resource for future breeding programs, for example, in supporting the selection of parental lines based on allelic and genotypic divergence. While the prediction of heterosis and the preparation of specific crosses were not experimentally addressed in this study, the data generated could provide the basis for such applications in future studies. Furthermore, these aspects remain crucial for the development of new molecular tools for protecting the rights of plant breeders and the interests of consumers of *Cannabis*. The implementation of these tools could thus contribute to ensuring the availability of certified varieties on the market, enabling consumers to access *Cannabis* products with higher transparency and security [34].

Moreover, the present study demonstrated the ability of the adopted genotyping method to assess the genetic structure of drug-type *Cannabis* samples collected from seed packages derived from the European distributors, and, consequently, compared the results with those of numerous previously analyzed samples, including both drug- and hemp-type plants, confirming its ability to compare data from different studies.

Notably, the results obtained revealed doubts regarding the quality and identity of some of the seeds currently present in the European market, which are probably mislabeled and/or cultivated using inappropriate production approaches, crossing standards or packaging strategies.

To conclude, the authors believe that the DNA-based labeling of varieties covered by patents represents a strong and reliable solution to protect the *Cannabis* market. This will undoubtedly help in managing intellectual property rights in the seed industry and protecting and consumers and professional farmers and derivative producers of *Cannabis*, thus reducing the risk of fraud and the illicit use of selected and registered varieties from the black market introduced by illegal organizations.

## Data Availability

The original contributions presented in this study are included in the article/Supplementary Materials. Further inquiries can be directed to the corresponding author.

## Supplementary Materials

The following supporting information can be downloaded at https://www.mdpi.com/article/10.3390/plants14193050/s1, Figure S1. ΔK chart indicating the most likely number of K for all (171) European Cannabis drug-type samples analyzed; Figure S2. ΔK chart indicating the most likely number of K for all (285) Cannabis samples; Figure S3. SSR loci distribution on *Cannabis sativa* chromosomes (A) Private alleles distribution among the 20 developed SSR loci; (B) SSR sites distribution among the 10 *Cannabis sativa* chromosomes; Table S1. Description of biological material reporting Cultivar/Variety ID, Origin, Cannabis Typology, main Characteristics, number of samples per variety/typology, and references are reported; Table S2. Primers organization in the 4 multiplexes for SSR loci amplification. Name, expected size, multiplex number, anchor, annealing temperature (°C), starting and ending genomic coordinates (chromosome ID is indicated in the first number present in the name of the marker locus), motif sequence with expected number of repetitions, and forward and reverse primer sequences are reported.

## References

[1] Kovalchuk I., Pellino M., Rigault P., van Velzen R., Ebersbach J., Ashnest J.R., Mau M., Schranz M.E., Alcorn J., Laprairie R.B. (2020). The Genomics of *Cannabis* and Its Close Relatives. Annu. Rev. Plant Biol..

[2] Yazici L. (2023). Optimizing plant density for fiber and seed production in industrial hemp (*Cannabis sativa* L.). J. King Saud Univ. Sci..

[3] Xu Y., Zhang J., Tang Q., Dai Z.G., Deng C.H., Chen Y., Cheng C.H., Yang Z.M., Zhang X.Y., Chen J.Q. (2024). Integrated metabolomic and transcriptomic analysis revealed the regulation of yields, cannabinoid, and terpene biosynthesis in *Cannabis sativa* L. under different photoperiods. S. Afr. J. Bot..

[4] Kalousek P., Holátko J., Schreiber P., Pluhácek T., Lónová K.S., Radziemska M., Tarkowski P., Vyhnánek T., Hammerschmiedt T., Brtnicky M. (2024). The effect of chelating agents on the Zn-phytoextraction potential of hemp and soil microbial activity. Chem. Biol. Technol. Agric..

[5] Malík M., Praus L., Kuklina A., Velechovský J., Janatová A.K., Klouček P., Mládek V., Tlustoš P. (2025). *Cannabis* yield and cannabinoid profile affected by plant nutrition and planting density. Ind. Crops Prod..

[6] Jin D., Henry P., Shan J., Chen J. (2021). Identification of Phenotypic Characteristics in Three Chemotype Categories in the Genus *Cannabis*. HortScience.

[7] Schultes R.E. (1970). Random Thoughts and Queries on the Botany of Cannabis.

[8] Small E., Cronquist A. (1976). A Practical and Natural Taxonomy for *Cannabis*. Taxon.

[9] Schultes R.E., Hofmann A. (1980). The Botany and Chemistry of Hallucinogens.

[10] Gerarde J. (1597). The Herball or General Historie of Plants.

[11] Clarke C. (1981). Marijuana Botany. An Advanced Study: The Propagation and Breeding of Distinctive Cannabis.

[12] Ming R., Bendahmane A., Renner S.S. (2011). Sex chromosomes in land plants. Annu. Rev. Plant Biol..

[13] Schultes R.E. (1982). Marihuana. The first twelve thousand years. J. Ethnopharmacol..

[14] Small E. (2015). Evolution and Classification of (Marijuana, Hemp) in Relation to Human Utilization. Bot. Rev..

[15] Lapierre E., de Ronne M., Boulanger R., Torkamaneh D. (2023). Comprehensive Phenotypic Characterization of Diverse Drug-Type *Cannabis* Varieties from the Canadian Legal Market. Plants.

[16] Hesami M., Pepe M., Alizadeh M., Rakei A., Baiton A., Jones A.M.P. (2020). Recent advances in *Cannabis* biotechnology. Ind. Crops Prod..

[17] Barcaccia G., Palumbo F., Scariolo F., Vannozzi A., Borin M., Bona S. (2020). Potentials and Challenges of Genomics for Breeding *Cannabis* Cultivars. Front. Plant Sci..

[18] Jin D., Henry P., Shan J., Chen J. (2021). Identification of Chemotypic Markers in Three Chemotype Categories of *Cannabis* Using Secondary Metabolites Profiled in Inflorescences, Leaves, Stem Bark, and Roots. Front. Plant Sci..

[19] Grassa C.J., Weiblen G.D., Wenger J.P., Dabney C., Poplawski S.G., Timothy Motley S., Michael T.P., Schwartz C.J. (2021). A new *Cannabis* genome assembly associates elevated cannabidiol (CBD) with hemp introgressed into marijuana. New Phytol..

[20] Lapierre E., Monthony A.S., Torkamaneh D. (2023). Genomics-based taxonomy to clarify *Cannabis* classification. Genome.

[21] Pancaldi F., Salentijn E.M.J., Trindade L.M. (2025). From fibers to flowering to metabolites: Unlocking hemp (*Cannabis sativa*) potential with the guidance of novel discoveries and tools. J. Exp. Bot..

[22] Cull A., Joly D.L. (2025). Development and validation of a minimal SNP genotyping panel for the differentiation of *Cannabis sativa* cultivars. BMC Genom..

[23] Zhang L., Yuan M., Tao A., Xu J., Lin L., Fang P., Qi J. (2015). Genetic Structure and Relationship Analysis of an Association Population in Jute (*Corchorus* spp.) Evaluated by SSR Markers. PLoS ONE.

[24] Gonzaga Z.J., Aslam K., Septiningsih E.M., Collard B.C.Y. (2015). Evaluation of SSR and SNP Markers for Molecular Breeding in Rice. Plant Breed. Biotech..

[25] Ghedina A., Galla G., Cadalen T., Hilbert J.L., Caenazzo S.T., Barcaccia G. (2015). A method for genotyping elite breeding stocks of leaf chicory (*Cichorium intybus* L.) by assaying mapped microsatellite marker loci. BMC Res. Notes.

[26] Adedze Y.M.N., Lu X., Fan W.Y., Zhang W.T., Yang X., Deng Z.J., Alam M.A., Xu G.L., Zhang L.H., Li W.H. (2024). Development of PCR-based markers associated with powdery mildew resistance using bulked segregant analysis (BSA-seq) in melon. Czech J. Genet. Plant.

[27] Tian F., Tian Y., Yu F., Qian J., Wang F., Li X., Li T., Zhang X., Huang D., Zhao X. (2025). Association analysis of the molecular characteristics and floral traits of Iris × germanica. Czech J. Genet. Plant.

[28] Soler S., Gramazio P., Figàs M.R., Vilanova S., Rosa E., Llosa E.R., Borràs D., Plazas M., Prohens J. (2017). Genetic structure of var. cultivars based on genomic SSR (gSSR) markers: Implications for breeding and germplasm management. Ind. Crops Prod..

[29] Zhang J., Yan J., Huang S., Pan G., Chang L., Li J., Zhang C., Tang H., Chen A., Peng D. (2020). Genetic Diversity and Population Structure of *Cannabis* Based on the Genome-Wide Development of Simple Sequence Repeat Markers. Front. Genet..

[30] Borin M., Palumbo F., Vannozzi A., Scariolo F., Sacilotto G.B., Gazzola M., Barcaccia G. (2021). Developing and Testing Molecular Markers in *Cannabis sativa* (Hemp) for Their Use in Variety and Dioecy Assessments. Plants.

[31] Alghanim H.J., Almirall J.R. (2003). Development of microsatellite markers in *Cannabis sativa* for DNA typing and genetic relatedness analyses. Anal. Bioanal. Chem..

[32] Gilmore S., Peakall R., Robertson J. (2003). Short tandem repeat (STR) DNA markers are hypervariable and informative in *Cannabis sativa*: Implications for forensic investigations. Forensic Sci. Int..

[33] Hsieh H.M., Hou R.J., Tsai L.C., Wei C.S., Liu S.W., Huang L.H., Kuo Y.C., Linacre A., Lee J.C. (2003). A highly polymorphic STR locus in *Cannabis sativa*. Forensic Sci. Int..

[34] Dufresnes C., Jan C., Bienert F., Goudet J., Fumagalli L. (2017). Broad-Scale Genetic Diversity of *Cannabis* for Forensic Applications. PLoS ONE.

[35] Gao C., Xin P., Cheng C., Tang Q., Chen P., Wang C., Zang G., Zhao L. (2014). Diversity analysis in *Cannabis sativa* based on large-scale development of expressed sequence tag-derived simple sequence repeat markers. PLoS ONE.

[36] Grassa C.J., Wenger J.P., Dabney C., Poplawski S.G., Motley S.T., Michael T.P., Schwartz C.J., Weiblen G.D. (2018). A complete *Cannabis* chromosome assembly and adaptive admixture for elevated cannabidiol (CBD) content. BioRxiv.

[37] Hillig K.W. (2005). Genetic evidence for speciation in *Cannabis* (Cannabaceae). Genet. Resour. Crop Evol..

[38] Lynch R.C., Vergara D., Tittes S., White K., Schwartz C.J., Gibbs M.J., Ruthenburg T.C., deCesare K., Land D.P., Kane N.C. (2017). Genomic and Chemical Diversity in *Cannabis*. Crit. Rev. Plant Sci..

[39] Sawler J., Stout J.M., Gardner K.M., Hudson D., Vidmar J., Butler L., Page J.E., Myles S. (2015). The Genetic Structure of Marijuana and Hemp. PLoS ONE.

[40] Soorni A., Fatahi R., Haak D.C., Salami S.A., Bombarely A. (2017). Assessment of Genetic Diversity and Population Structure in Iranian *Cannabis* Germplasm. Sci. Rep..

[41] Fisarova L., Surinova M., Jarosova A., Krejcik J., Vosatka M. (2024). Evidence of the Ability of Microsatellite Method to Distinguish *Cannabis* Strains with High Cannabinoid Content. Cannabis Cannabinoid Res..

[42] Cheng Y.C., Houston R. (2025). The development of two fast genotyping assays for the differentiation of hemp from marijuana. J. Forensic Sci..

[43] Vyhnanek T., Nevrtalova E., Bjelkova M., Balgova B. (2020). SSR loci survey of technical hemp cultivars: The optimization of a cost-effective analyses to study genetic variability. Plant Sci..

[44] Hu Z.G., Guo H.Y., Hu X.L., Chen X., Liu X.Y., Guo M.B., Zhang Q.Y., Xu Y.P., Guo L.F., Yang M. (2012). Genetic diversity research of hemp (*Cannabis sativa* L) cultivar based on AFLP analysis. Plant Gene. Res..

[45] Zhang Q., Chen X., Guo H., Trindade L.M., Salentijn E.M.J., Guo R., Guo M., Xu Y., Yang M. (2018). Latitudinal Adaptation and Genetic Insights Into the Origins of *Cannabis sativa* L. Front. Plant Sci..

[46] Benkirane C., Charif M., Müller C.M., Taaifi Y., Mansouri F., Addi M., Bellaoui M., Serghini-Caid H., Elamrani A., Abid M. (2023). Population structure and genetic diversity of Moroccan *Cannabis* (*Cannabis sativa* L.) germplasm through simple sequence repeat (SSR) analysis. Genet. Resour. Crop Evol..

[47] Rull V. (2022). Origin, early expansion, domestication and anthropogenic diffusion of *Cannabis*, with emphasis on Europe and the Iberian Peninsula. Perspect. Plant Ecol..

[48] Groom Q. (2014). R.C. Clarke & M.D. Merlin (2013)–*Cannabis*: Evolution and Ethnobotany. Plant Ecol. Evol..

[49] Clarke R.C., Merlin M.D. (2016). Domestication, Breeding History, Present-day Genetic Diversity, and Future Prospects. Crit. Rev. Plant Sci..

[50] Borin M. (2024). Overcoming Challenges in *Cannabis sativa* Breeding Research with Conventional and Biotechnological Tools. Ph.D. Thesis.

[51] Schuelke M. (2000). An economic method for the fluorescent labeling of PCR fragments. Nat. Biotechnol..

[52] Falush D., Stephens M., Pritchard J.K. (2003). Inference of population structure using multilocus genotype data: Linked loci and correlated allele frequencies. Genetics.

[53] Evanno G., Regnaut S., Goudet J. (2005). Detecting the number of clusters of individuals using the software STRUCTURE: A simulation study. Mol. Ecol..

[54] Yeh F.C., Boyle T.J.B. (1997). Population Genetic Analysis of Codominant and Dominant Markers and Quantitative Traits. Belg. J. Bot..

[55] Levene H. (1949). On a matching problem arising in genetics. Ann. Math. Stat..

[56] Nei M. (1978). Estimation of average heterozygosity and genetic distance from a small number of individuals. Genetics.

[57] Rohlf F.J. (2009). NTSYSpc: Numerical Taxonomy and Multivariate Analysis System Ver. 2.2.

[58] Jombart T. (2008). adegenet: A R package for the multivariate analysis of genetic markers. Bioinformatics.

[59] Jombart T., Ahmed I. (2011). adegenet 1.3-1: New tools for the analysis of genome-wide SNP data. Bioinformatics.

[60] Wickham M.H. (2016). ggplot2. Use R!.

[61] Kamvar Z.N., Brooks J.C., Grunwald N.J. (2015). Novel R tools for analysis of genome-wide population genetic data with emphasis on clonality. Front. Genet..

[62] Peakall R., Smouse P.E. (2012). GenAlEx 6.5: Genetic analysis in Excel. Population genetic software for teaching and research—An update. Bioinformatics.

[63] Botstein D., White R.L., Skolnick M., Davis R.W. (1980). Construction of a genetic linkage map in man using restriction fragment length polymorphisms. Am. J. Hum. Genet..

[64] Scariolo F., Palumbo F., Farinati S., Barcaccia G. (2023). Pipeline to Design Inbred Lines and F1 Hybrids of Leaf Chicory (Radicchio) Using Male Sterility and Genotyping-by-Sequencing. Plants.

[65] Mufti S., Afroza B., Bhat R., Mushtaq S., Nabi A. (2017). DUS testing for plant variety protection. Ann. Agri Bio Res..

[66] Hall J., Bhattarai S.P., Midmore D.J. (2012). Review of Flowering Control in Industrial Hemp. J. Nat. Fibers.

[67] Vergara D., Huscher E.L., Keepers K.G., Pisupati R., Schwabe A.L., McGlaughlin M.E., Kane N.C. (2021). Genomic Evidence That Governmentally Produced *Cannabis sativa* Poorly Represents Genetic Variation Available in State Markets. Front. Plant Sci..

[68] de Oliveira Pereira Ribeiro L., Avila E., Mariot R.F., Fett M.S., de Oliveira Camargo F.A., Alho C.S. (2020). Evaluation of two 13-loci STR multiplex system regarding identification and origin discrimination of Brazilian *Cannabis sativa* samples. Int. J. Leg. Med..

[69] Palumbo F., Galla G., Barcaccia G. (2017). Developing a Molecular Identification Assay of Old Landraces for the Genetic Authentication of Typical Agro-Food Products: The Case Study of the Barley ‘Agordino’. Food Technol. Biotechnol..

