# Supplementary figures and images for: Genotyping of Commercial European Cannabis Seeds Based on Multiple Mapped Marker Loci: A Comparative Study of Drug and Hemp Varieties
Source: Plants (Basel). 2025 Oct 2;14(19):3050. doi: 10.3390/plants14193050 (PMC12526286; doi:10.3390/plants14193050)

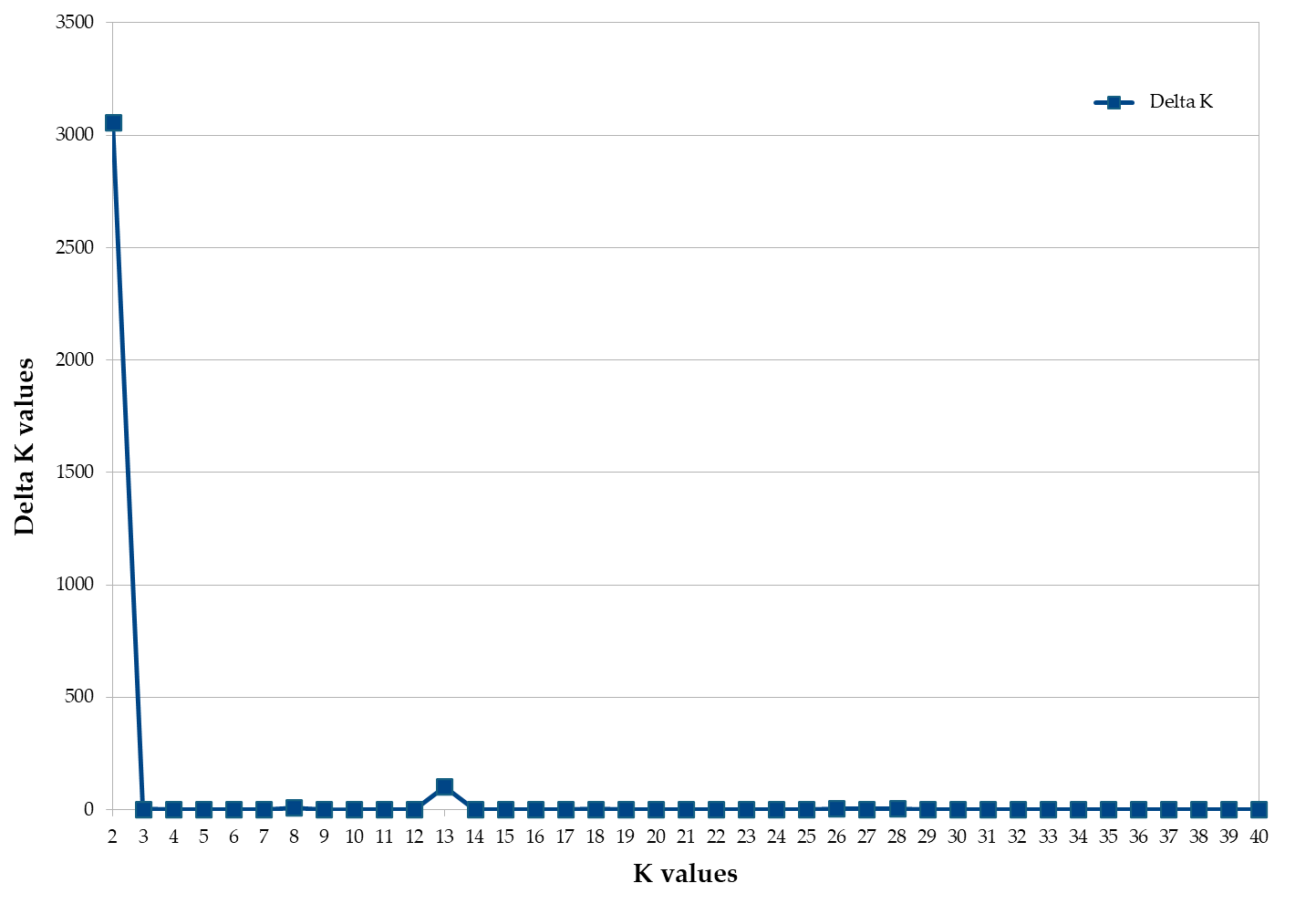


**Figure S2:** ΔK chart indicating the most likely number of K for all (285) *Cannabis* samples.

Supplement: Supplementary file 1 [file plants-14-03050-s001.zip › Supplementary_Proof/Figure_S2_proof-edited.docx]
